# Opioids and the Kidney: A Compendium

**DOI:** 10.34067/KID.0000000000000291

**Published:** 2023-11-06

**Authors:** Steven Didik, Daria Golosova, Biyang Xu, Alexander Staruschenko

**Affiliations:** 1 Department of Molecular Pharmacology and Physiology, University of South Florida, Tampa, Florida; 2 James A. Haley Veteran's Hospital, Tampa, Florida; 3 Department of Physiology, Medical College of Wisconsin, Milwaukee, Wisconsin; 4 Hypertension and Kidney Research Center, University of South Florida, Tampa, Florida

**Keywords:** opioid physiology, opioid receptors, opioids and kidney damage, podocytopathy

## Abstract

Opioids are a class of medications used in pain management. Unfortunately, long-term use, overprescription, and illicit opioid use have led to one of the greatest threats to mankind: the opioid crisis. Accompanying the classical analgesic properties of opioids, opioids produce a myriad of effects including euphoria, immunosuppression, respiratory depression, and organ damage. It is essential to ascertain the physiological role of the opioid/opioid receptor axis to gain an in-depth understanding of the effects of opioid use. This knowledge will aid in the development of novel therapeutic interventions to combat the increasing mortality rate because of opioid misuse. This review describes the current knowledge of opioids, including the opioid epidemic and opioid/opioid receptor physiology. Furthermore, this review intricately relates opioid use to kidney damage, navigates kidney structure and physiology, and proposes potential ways to prevent opioid-induced kidney damage.

## Introduction

Opioids are a popular class of drugs used for centuries both medically and recreationally. The poppy plant *Papaver somniferum* contains the primary active compounds used to produce naturally derived and synthetic analgesics.^1^ For clarification, an *opioid* is any chemical compound not derived from plants and synthesized in a laboratory, in which more than 500 have been produced.^2^ Examples of opioids include the synthetic phenylpiperidines meperidine and fentanyl and the semisynthetic derivatives, oxycodone and hydromorphone^1,3^ An *opiate* is a compound extracted directly from plant matter.^2^ Examples of opiates include the alkaloids morphine and codeine.^3,4^ For the remainder of this review, the term opioids will be used as a blanket term to describe both opioids and opiates.

Both opioids and opiates bind to opioid receptors.^4,5^ Opioid receptors are conserved G protein–coupled receptors populated throughout the body, spanning the central and peripheral nervous systems, organs, and various cell types.^6^ Numerous G protein–coupled receptors are expressed in the kidneys as well.^7^ Multiple opioid receptors exist, and this review will discuss the three classical opioid receptors: kappa receptors (KORs), mu receptors (MORs), and delta receptors (DORs) and their function, location, and expression in the kidney.^6,8^

The primary rationale for opioid use is pain management.^1,4,5^ Chronic pain affects approximately 100 million adults in the United States.^9^ Medical opioid use for acute pain, end-of-life care, and cancer pain is invaluable for clinicians and patients.^10^ The pain relief benefits and euphoric properties of opioids make the drug also highly attractive for recreational use. Overprescription and recreational opioid use contribute to 91 deaths due to overdose per day in the United States.^9^ This pattern of use is responsible for the development of the opioid epidemic in the United States and across the world.^2,10^

Opioid use can have injurious effects on organs in the body, including the kidney. This review describes opioid physiology in relation to kidney dysfunction. In addition, this mini-review provides ideas for novel therapeutic interventions to combat opioid-induced kidney damage.

## Opioid Physiology

### Primary Physiologic Mechanism

**Figure 1:**
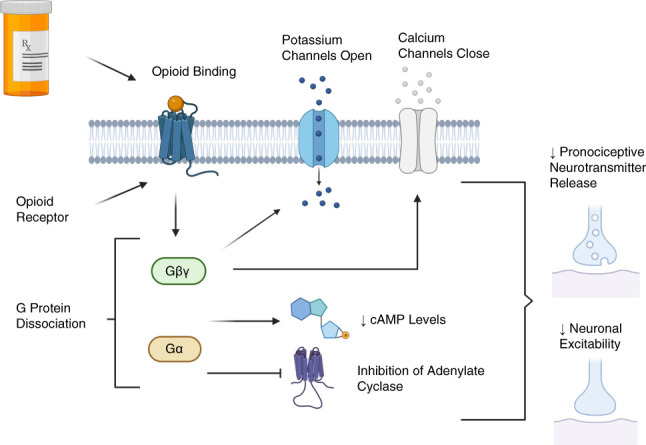
**Fundamental opioid mechanism of action in analgesia.** Opioid/opioid receptor binding induces GPCR dissociation into G*γβ* and G*α* subunits. The G*γβ* subunit closes calcium channels and opens potassium channels. The G*α* subunit decreases cAMP levels and inhibits adenylate cyclase. These actions lead to decreased discharge of pronociceptive neurotransmitters and decreased neuronal excitability, decreasing the sensation of pain. cAMP, cyclic adenosine monophosphate; GPCR, G protein-coupled receptor.

Both natural and synthetic opioids bind to opioid receptors (Table 1).^11–14^ Opioid receptors are widely distributed throughout the body and induce a myriad of effects. The primary effect of opioids is analgesia.^15^ The classical analgesic pathway of opioids leads to decreases in excitability and transmission of neuronal pain pathways.^4^ As an opioid binds to an opioid receptor, intracellular coupled G-proteins dissociate into their G*γβ* and G*α* subunits.^3^ The G*γβ* subunit opens potassium channels and closes voltage gated calcium channels.^3^ The G*α* subunit inhibits adenylate cyclase and decreases cyclic adenosine monophosphate levels.^3^ As a result, neuronal excitability decreases, as well as the discharge of pronociceptive neurotransmitters, creating the decreased sensation of pain (Figure 1).^3^

**Table 1:** Opioid receptor location, function, common agonists and antagonists, and endogenous peptides

Receptor	Location	Function	Common Agonists	Common Antagonists	Endogenous Peptide
Delta (δ)	• Neocortex • Olfactory bulb • Nucleus accumbens • Amygdala • CNS, PNS presynaptic/postsynaptic neurons • Kidney	• Inhibit neurotransmitter release • Respiratory regulation • Antidepressive effects • Analgesia	• Oxycodone • Etorphine • TAN-67	• Naltriben • SB205588 • Naltrindole • TIPP-ψ	• (Leu)-enkephalin • (Met)-enkephalin • β-endorphin
Kappa (κ)	• Striatum • Nucleus accumbens • Podocytes • Amygdala • Claustrum • CNS, PNS presynaptic/postsynaptic neurons	• Dysphoria production • Sedation • Diuresis • Analgesia	• Nalfurafine • Salvinorin-A • Collyboldie • TRK820 • U50,488 • U69,593	• JNJ-67953964 • TENA • GNTI • Nor-BMI • UPHIT • DIPPA • BTRX-335140	• Metorphamide • Dynorphin A • Dynorphin B
Mu (µ)	• Medulla • Mesolimbic dopaminergic neurons • Mesenteric plexus • Immune cells • CNS, PNS presynaptic/postsynaptic neurons	• Respiratory depression • Pleasure/Reward activation • Gut motility • Immune suppression	• Morphine • Loperamide • Buprenorphine • Tramadol • Methadone • Fentanyl • Remifentanil	• Naloxone • Naltrexone • Cyprodime • *β*-FNA • DOC-CAM • MET-CAMO • Naloxonazine	• Endomorphin-1 • Endomorphin-2 • *β*-endorphin • Dermorphin

CNS, central nervous system; PNS, peripheral nervous system; TIPP-c; H-Tyr-Tic [CH2NH]-Phe-Phe-OH; TENA, triethyleneglycol-naltrexamine; BMI, body mass index; GNTI, 5’-Guanidinonaltrindole; UPHIT, (1S,2S)-trans-2-Isothiocyanato-4,5-dichloro-N-methyl-N-[2-(1-pyrrolidinyl)cyclohexyl] benzeneacetamide; β-FNA, β-funaltrexamine; DOC-CAM, deoxyclocinnamox; MET-CAMO, 5β-methyl-14β-(*p*-nitrocinnamoylamino)-7,8-dihydromorphinone.

### Opioid Receptors

Rigorous research has been conducted to gain further understanding of the function and signaling pathways of opioid receptors. The three classical opioid receptors are delta (δ), kappa (κ), and mu (µ) receptors (DORs, KORs, and MORs, respectively). The receptors are located across the central and peripheral nervous system, within different cell types, and throughout the body.^14^

DORs are most anatomically populated at the neocortex, olfactory bulb, nucleus accumbens, and amygdala.^14,16^ The primary function of DOR lies in their presynaptic location, where they block neurotransmitter release.^14^ Furthermore, DOR can be found modulating neurotransmitter release in respiratory neurons, serving a purpose in respiratory pattern production.^14^ DOR activation also produces antidepressive effects.^14,17^ Studies have revealed an increase in anxiety and depression when the *Oprd 1* gene (encoding gene for DORs) was knocked out in mice.^17^

KORs serve functions independent of other opioid receptors, including analgesia, diuresis, and brain reward function.^14,18^ KORs are coupled to calcium channels on presynaptic terminals and are found in the nucleus accumbens, claustrum, amygdala, hypothalamic nuclei, and, notably, podocytes in the kidney.^18^ KORs have a role in brain reward operation where activation produces dysphoria by inhibiting dopamine release in the striatum.^19^ KORs on presynaptic terminals coupled to calcium channels inhibit calcium current flow, thus inhibiting neurotransmitter (dopamine) release.^18^ KORs also serve a role in analgesia.^14^ Finally, KOR activation produces analgesia and sedation independent of increases in respiratory depression and heart rate.^14^ This characteristic makes KOR an excellent target for therapeutic investigation.

MORs are the clinical target for prescribed opioids.^20^ The principal role of the MOR is analgesia.^20^ Located presynaptically and postsynaptically spanning the nervous system, MORs decrease neuronal calcium influx and increase neuronal potassium influx, mitigating neuronal transmission and alleviating pain.^21^ MORs are also located in brain reward circuits.^14^ In the brain reward pathway, MORs can be found in mesolimbic dopaminergic neurons, the nucleus accumbens, amygdala, hypothalamus, brainstem nuclei, and locus coeruleus.^22^ MORs in brain reward circuits are involved in pleasure, reward, reinforcement, and addictive states.^14,20^

#### DOR, KOR, and MOR Expression in the Kidney

Studies have been conducted to identify and quantify opioid receptor gene expression in rodent and human kidneys. Conflicting evidence exists regarding opioid receptor gene expression in the human kidney. In one analysis, absolute quantitative RT-PCR detected no MOR gene expression, a small amount of mRNA KOR gene expression, and high DOR gene expression in the human kidney.^23^ Another *in vitro* analysis in human podocytes revealed high levels of MOR and KOR and almost no DOR gene expression.^24^ Confirmational immunofluorescence was performed on mouse kidneys, and the results aligned with the human RT-PCR findings in this study.^24^ RT-PCR performed in rat kidneys reported low levels of KOR and DOR gene expression and moderate levels of MOR gene expression.^25^ Work performed by our group revealed KOR expression in rat and human podocytes using immunohistochemistry and RT-PCR.^26^

### Nonanalgesic Mechanisms of Opioids

Opioids produce effects independent of analgesia. Opioids act on mood/reward pathways, depress respiration, and induce diuresis.^17,21,27^ In mood/reward pathways, the pleasure sensation of opioid use is a result of gamma-aminobutyric acid (GABA) inhibition by MORs in neuronal reward pathways.^28^ GABA is the main neurotransmitter for inhibition in the central nervous system.^29^ Inhibition of GABA results in dopaminergic neuron hyperactivity in the nucleus accumbens and excess dopamine production.^28^ This produces the intense pleasure experience associated with opioid use.^28^

Opioid-induced respiratory depression is the leading cause of all opioid-related deaths.^27^ The MORs located in the medulla are the principal receptors involved in respiratory depression.^27^ The medulla comprises a network of synchronized neurons responsible for producing breathing, delicate to opioid use.^27^ This network contains the preBötzinger complex.^27^ Studies show that disruptions of the preBötzinger complex can lead to respiratory failure.^27^

KOR activation induces water diuresis *via* renal sympathetic nerve stimulation, decreasing antidiuretic hormone (ADH) action in the kidney and decreasing ADH release by the brain.^30^ In a study involving KOR agonists in normally hydrated rats, 2-Methoxymethyl-salvinorin, nalfurafine, and U50,488H, all increased free water clearance and urine volume compared with controls.^30^ In humans, administration of KOR agonists MR 2034, spiradoline, and MR 2033 increased urine volume output.^31^ The diuresis effect of KOR agonists/KOR activation has been studied in conditions where diuresis could serve therapeutic purposes.^30^ In rats with hypertension, one study confirmed that administration of the KOR agonist U50,488H produced declines in mean arterial pressure, decreased plasma ADH, and induced diuresis when compared with normotensive rats.^32^ Other opioid receptors play a role in diuresis. Administration of the central selective MOR agonist demorphin led to an increased diuresis and decreased sodium excretion without changing plasma flow, GFR, or the sympathetic outflow to the kidneys.^33^ DOR agonist BW373U86 acutely increased diuresis and natriuresis through autonomic nervous system regulation.^34^

## Opioids and the Kidney

### Foundational Kidney Structure and Function

**Figure 2:**
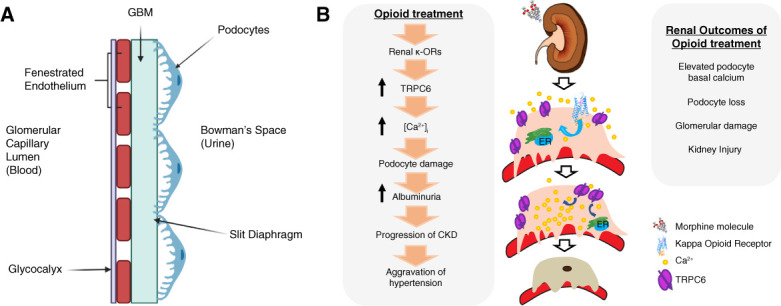
**Proposed mechanism of glomerular injury through the kappa opioid receptors.** (A) Components of the GFB. Three major cell types comprise the barrier: the fenestrated endothelium, GBM, and podocytes creating the ultrafiltration component of the nephron. (B) Podocytopathy due to opioid use. Opioids bind to kappa opioid receptors on podocytes. Binding results in TRPC6 channel activation and a significant increase in calcium influx. Increased amounts of calcium induce podocytopathy, leading to the progression of kidney disease and exacerbation of hypertension. GBM, glomerular basement membrane; GFB, glomerular filtration barrier; TRPC6, transient receptor potential cation 6.

The kidneys are organs responsible for waste removal, urine production, BP regulation, and the maintenance of minerals, water, and salts.^35^ The functional unit of the kidney is the nephron, which comprises the glomerulus.^35,36^ The glomerulus is the main filtering unit of the kidney.^35^ The glomerular filtration barrier (GFB) is formed by three major cell types: podocytes, glomerular basement membrane (GBM), and the fenestrated endothelium (Figure 2A).

### Podocyte Structure and Physiology

It is necessary to describe the cytoarchitecture and physiology of the podocyte because studies have linked opioid use to podocytopathy.^24,26^ Opioids can disrupt podocyte structure and function^26^ (Figure 2B). Podocyte dysfunction is the catalyst for the development of kidney diseases, including FSGS, CKD, and hypertension.^37–41^ Podocytes are atypical epithelial cells.^38,42^ The podocyte comprises three structures: foot processes, the cell body, and the extending processes.^43^ The cell body is oriented toward the Bowman's space. The extending processes continue from the cell body, which then divide into foot processes.^43^ Foot processes intertwine with neighboring foot processes and the GBM, forming the end-stage of filtration slit diaphragm of the GFB.^42^ For proper adhesion and communication with the GBM, the slit diaphragm uses proteins including nephrin, synaptopodin, CD2AP, NEPH-1, ZO-1, and podocin.^44,45^ Podocyte effacement is an injurious consequence that can occur during a disruption in the anatomical actin cytoskeleton and support proteins forming the connection to the GBM.^46,47^

### Opioids and Kidney Pathology

Kidney damage is a dangerous side effect arising from chronic opioid use, and kidney disease has been shown to increase concurrently with opioid use.^48^ Novel medical approaches are needed to protect the kidney from opioid-induced insults.

**Figure 3:**
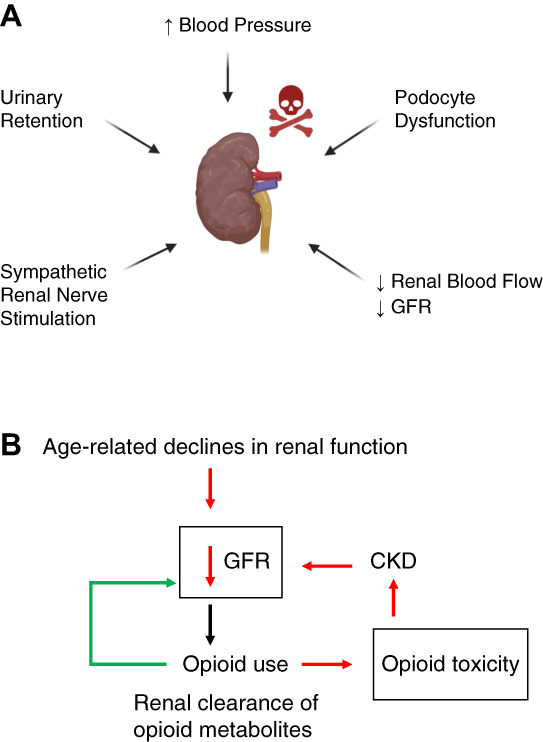
**Proposed mechanism of renal injury.** (A) Opioid-induced mechanisms leading to kidney damage. Opioid use contributes to increases in BP, podocytopathy, decreases in renal blood flow and GFR, stimulation of the renal nerves, and urinary retention. (B) Roadmap of opioid-mediated mechanisms leading to kidney damage. Opioid use and poor renal clearance of opioid metabolites decrease GFR, contributing to local and systemic opioid toxicity and further kidney damage. Opioid use, along with age-related declines in renal function, also contributes to local and systemic opioid toxicity.

Opioids are metabolized in the liver, and the kidney-sensitive metabolites are processed through the kidney.^49^ Chronic opioid use leads to the local and systemic accumulation of noxious metabolites.^50^ Prolonged opioid use and the toxic metabolite aggregation catalyze mechanisms leading to kidney damage, including increased BP, podocyte dysfunction, urinary retention, decreased renal blood flow and GFR, and sympathetic renal nerve stimulation (Figure 3).^50^

The increased sympathetic renal nerve stimulation by opioid misuse causes vasoconstriction, increased BP, decreased blood flow, and renal ischemia.^50,51^ Increased BP and decreased oxygen quantity occur at the glomerulus inducing glomerular damage and podocytopathy, leading to kidney dysfunction and increases in BP.^26^

Work by our group has demonstrated that podocyte KOR activation opens transient receptor potential cation 6 (TRPC6) channels increasing calcium influx into the cell, causing podocyte dysfunction (Figure 2B).^26^ Our study used immortalized human podocytes, isolated human and rat glomeruli, and Dahl salt-sensitive rats. To observe calcium influx exclusivity by TRPC6, administration of SAR7334 (TRPC6 inhibitor) with BRL52537 (KOR agonist) showed complete inhibition of the BRL52538-induced calcium response in human podocytes, captured by radiometric confocal fluorescent microscopy.^26^ Dahl salt-sensitive rats exhibited increased mean arterial pressure and microalbuminuria after chronic BRL52537 treatment. Podocytes isolated from hypertensive animals also exhibited a steady increase in total calcium influx following BRL52537 administration.^26^ Therefore, podocytopathy due to opioid use exacerbates the progression of hypertension and furthers kidney damage, ultimately leading to diseases, such as FSGS.^26^

Further evidence has been substantiated involving opioid use and podocytopathy. Morphine has been shown to induce podocyte effacement in mouse models and decrease slit diaphragm proteins in human podocytes *in vitro*.^24^ In this study, increased albuminuria was observed on high-dose morphine administration in mice.^24^ Western blot of mouse kidney tissue lysates also showed declines in podocyte proteins nephrin and synaptopodin.^24^ These results were consistent with western blot analysis of human podocytes treated with 10^−8^ or 10^−6^ M morphine for 24 hours, which revealed declines in nephrin, synaptopodin, CD2AP, and podocin.^24^

Clinical studies in human patients investigating opioid-induced podocytopathy merit further investigation. Podocytopathy in human patient population can contribute to an array of diseases such as FSGS, nephrotic syndrome, and ESKD.^45^ Clinical insight into the degree of podocyte damage caused by opioid use could reveal mechanistic patterns and correlate to a number of kidney diseases.

In addition to podocytes, other glomerular cells can be affected by opioid misuse. Mesangial cells are responsible for the proper clearance of materials before the GFB, and over-proliferation of mesangial cells contributes to glomerulopathy.^38,52^ Morphine use has been shown to induce glomerulopathy *via* induction of mesangial cell proliferation.^44^ In this study, histopathology of morphine-treated kidneys revealed mesangial cell area increases and glomerular enlargement.^44^

In chronic opioid use, the anticholinergic character of various opioids on the MOR induces urinary retention.^50^ Here, sensory nervous function is inhibited, and the micturition reflex is impaired. The consequence of urinary retention is the generation of permanent renal scarring, due to prolonged high levels of reverse flow pressure.^51^

To mitigate the detrimental effects of opioid use described, attempts to establish safe opioid prescribing protocols have been made by the Center for Disease Control (CDC).^53,54^ However, the evolving nature of the opioid crisis makes setting a gold standard of care highly complex. First, the CDC recommends nonopioid alternatives for pain management, such as the Mindfulness-Oriented Recovery Enhancement behavioral protocol before initiating opioid therapy.^54–56^ If opioid therapy is needed, the CDC recommends beginning with a regimen of the lowest dose of instant-release opioids with the long-term goal of treatment cessation.^55^ The effects of chronic opioid use on the organs remain unknown and warrant further investigation and novel prescribing protocols.

Therapeutic modalities mitigating the detrimental effects of opioids on the kidney warrant further investigation. Novel approaches to mitigating kidney damage include the development of a bivalent or bifunctional medication targeting podocyte KOR. In addition, a compound capable of simultaneous administration with opioids could modulate podocyte KOR and TRPC6 channels.

## Dialysis and Opioid Use

Chronic pain is a major concern in patients with ESKD. Approximately half of the patients receiving hemodialysis report chronic pain, and more than 60% of dialysis patients are prescribed, at minimum, one opioid per year.^57,58^ Of these prescriptions, 25% are higher doses than the recommended range.^59^ The most frequently prescribed opioids for hemodialysis patients from 2006 to 2010 were oxycodone and hydrocodone.^57^ Most notably, large analyses of opioid administration and patient outcomes remarkably confirmed a direct relationship between opioid use and mortality in dialysis patients.^57^ Furthermore, analyses have shown that chronic opioid use in the dialysis population generates many unfavorable side effects, leading to long-term hospitalizations and dialysis discontinuation.^57^

Dialysis treatment and renal failure heavily alter drug pharmacokinetics and pharmacodynamics, increasing the likelihood of detrimental side effects.^59^ Poor renal clearance contributes to local and systemic toxic metabolite accumulation.^59^ Metabolite accumulation is further exacerbated by the reduced hepatic metabolic capabilities, including the effects of kidney failure on hepatic enzymes from the cytochrome P450 (CYP) superfamily.^59,60^ Patients with cytochrome P450 (CYP) polymorphism might have a significantly altered opioid concentration and could be at increased risk of drug interactions with CYP inducers or inhibitors.^61^ Excess accumulation of metabolites can lead to hypotension, central nervous system depression, seizures, respiration depression, and death in the hemodialysis population.^59^ Examples of medications with poor renal clearance in hemodialysis patients are fentanyl, methadone, morphine, and oxycodone.^59^ However, methadone and fentanyl, with 25%–50% dose reduction, and buprenorphine are considered relatively safe opioids in the management of pain in CKD under hemodialysis.^62–65^

CKD-associated pruritus (CKD-aP), a condition of itch lasting >6 weeks in patients with CKD without correlation to the level of uremia, is a common comorbidity in dialysis patients.^66,67^ Recently, the US Food and Drug Administration approved difelikefalin, a peripherally restricted and selective KOR agonist for moderate-to-severe CKD-aP.^68^ Majority of trials show significant improvement of the CKD-aP conditions; however, no studies reported analyses of kidney function during the treatment.^69,70^ Phase 3 clinical trials are currently underway for the safety and efficiency of difelikefalin in patients with CKD (Trial registration number NCT05342623).

Opioid analgesics in CKD should be adjusted to the degree of renal impairment at the onset and throughout treatment. Further research is needed regarding the safety and risk-benefit ratio of opioid administration in hemodialysis patients. Before initiating opioid treatment protocol, nonopioid therapeutics could be considered for pain management, including cognitive behavioral therapy, acetaminophen, topical nonsteroidal anti-inflammatories, and gabapentin.^57,59^

## Potential Strategies Targeting Podocyte KORs to Mitigate Kidney Damage

Podocyte KORs are a potential therapeutic target for the prevention of opioid-induced kidney damage. Attenuating the detrimental effects of opioids is a state-of-the-art approach to the development of effective organ protection. Novel targeting strategies include the simultaneous administration of a KOR antagonist, locationally contained agonists, and the development of bivalent and bifunctional medications.

### The Simultaneous Administration of KOR Antagonists

The synchronous administration of opioids and an effective KOR antagonist could concentration-dependently attenuate the rate of deleterious calcium influx at the TRPC6 channel on the podocyte, preserving podocyte viability. Promising KOR antagonists that warrant further investigation in their therapeutic potential to combat kidney disease are NMRA-140 (formerly BTRX-335140) and JNJ-67953964.

NMRA-140 is a novel and highly attractive KOR antagonist currently under clinical trial investigation because of its short-acting block of KOR and antidepressive function.^71,72^ Short-acting antagonists are beneficial in decreasing damaging proinflammatory signaling pathways, normally activated by long-lasting antagonists.^71^ The high KOR selectivity (8125 versus DOR, 138 versus MOR) reduces off-target side effects, and its short action time mimics traditional medications.^71,73^ Studies examining the potency of BTRX-335140 conclude high potency and positive measures of target engagement.^71^ The distinct properties of MNRA-140 afford the medication extensive examination in its protective role in opioid-induced kidney damage.

Another KOR antagonist of interest in the prevention of opioid-induced kidney damage is JNJ-67953964. JNJ-67953964 also boasts an advantageous short action time, is well tolerated for human use, and has a high KOR affinity.^74^ Studies have demonstrated improvements in depressive behavior on JNJ-67953964 administration.^75^ The unique characteristics of JNJ-67953964 affirm the medication as an excellent candidate for investigating its simultaneous use with opioids in mitigating opioid-induced kidney damage.

### Locationally Contained Agonists

Recent progress has been made in structurally modifying KOR agonists to confine their location of action to designated locations in the body.^76^ In this approach, alterations to agonist structure could restrict agonist access to the podocyte. Ideally, this modality would induce analgesia while simultaneously serving a protective role in the kidney.

### Bivalent and Bifunctional Medications

Bifunctional medications are drugs with a common core capable of acting on multiple targets.^14^ Bivalent medications structurally incorporate two pharmacophoric regions united by a liker, which permits dual-purpose action of the medication.^14^ A medication of these types could have both antagonist and antagonist properties. Supporting evidence has shown bivalent compounds with varying linker chain lengths exhibit increased selectivity and potency compared with their respective individual compounds.^77^ In the case of kidney protection, the theoretical compound could elicit KOR agonist analgesic effects at one pharmacophore while possessing a distinct pharmacophoric region functioning as a TRPC6 antagonist. In this case, calcium influx could be properly regulated on KOR agonist administration, preserving podocyte function. The efficacious design of novel bivalent and bifunctional medications could serve as breakthrough treatment options for kidney and other organ protection.

In conclusion, opioid misuse is a major threat to society. With prescription mishandling and illicit use surging, proper opioid legislative action and the accompanying detrimental physiological opioid effects require immediate attention. A comprehensive understanding of pain biology and opioid pharmacology could lead to the development of necessary deviations from the injurious classical use of opioids. Novel treatment options, including medications to be taken concurrently with opioids, bifunctional and bivalent medications, and locally contained opioid receptor agonists, need to be further elucidated. These modalities could prevent damage to opioid-induced kidney damage and reduce the incidence of deaths due to opioid use, combating the opioid epidemic.
